# Regenerating Articular Tissue by Converging Technologies

**DOI:** 10.1371/journal.pone.0003032

**Published:** 2008-08-21

**Authors:** Lorenzo Moroni, Doreen Hamann, Luca Paoluzzi, Jeroen Pieper, Joost R. de Wijn, Clemens A. van Blitterswijk

**Affiliations:** 1 Institute for BioMedical Technology (BMTI), University of Twente, Enschede, The Netherlands; 2 Mechanical Engineering Institute, Universitá Politecnica delle Marche, Ancona, Italy; 3 Isotis OrthoBiologics S.A., Irvine, California, United States of America; 4 Rizzoli Orthopaedic Institute, Muscolo-Skeletal Cell and Tissue Bank, Bologna, Italy; University of Sheffield, United Kingdom

## Abstract

Scaffolds for osteochondral tissue engineering should provide mechanical stability, while offering specific signals for chondral and bone regeneration with a completely interconnected porous network for cell migration, attachment, and proliferation. Composites of polymers and ceramics are often considered to satisfy these requirements. As such methods largely rely on interfacial bonding between the ceramic and polymer phase, they may often compromise the use of the interface as an instrument to direct cell fate. Alternatively, here, we have designed hybrid 3D scaffolds using a novel concept based on biomaterial assembly, thereby omitting the drawbacks of interfacial bonding. Rapid prototyped ceramic particles were integrated into the pores of polymeric 3D fiber-deposited (3DF) matrices and infused with demineralized bone matrix (DBM) to obtain constructs that display the mechanical robustness of ceramics and the flexibility of polymers, mimicking bone tissue properties. Ostechondral scaffolds were then fabricated by directly depositing a 3DF structure optimized for cartilage regeneration adjacent to the bone scaffold. Stem cell seeded scaffolds regenerated both cartilage and bone *in vivo*.

## Introduction

Osteochondral defects are typically derived from congenital diseases or traumatic events in young patients and from osteoarthritis in old individuals. This results in associated pain, joint stiffness and instability, and often leads to the replacement of joint functionality with prosthesis. Although bone tissue has the capacity to regenerate, bone repair is impaired in many pathological situations. Furthermore, cartilage has a poor capacity to regenerate itself due to its avascular nature and to its intrinsic composition. Immobilization of patients is often the last stage of these degenerative and painful processes. Therefore, there is a critical need to develop technologies to promote bone and chondral healing. Autografts–tissues transplanted from one part of the body to another in the same patient, namely here bones or osteochondral plugs–are the most common treatments for osteochondral defects. However, clinical use involves some difficulties including septic complications, viral transmission, and morbidity in the location where tissue is harvested [1], [2]. A possible solution in terms of availability would be the use of allografts–tissues transplanted from one part of a donor's body to the same or another part of a recipient patient. Yet, these may be associated to risks of disease transmission, and complications in shaping. Other significant additional limitations of allografts are delay in remodeling of the bony part by the host and a lack of integration with the surrounding chondral tissue. Furthermore, in the case of very large defects the allograft may remain in the implant site throughout the patient's life, creating an area more prone to fracture or infection [3]–[5].

These issues have justified the development of 3D scaffolds employed for tissue regeneration, which are an attractive alternative when used alone or in combination with cells to restore the joint functionality [5]–[7]. Osteochondral scaffolds typically comprise a cartilage and a bone compartment. Chondral scaffolds are generally formed by polymeric foams or textile meshes [8]–[12], while bone substitutes include either biomaterials mimicking the composition of bone, i.e. calcium phosphate ceramics simulating the bone mineral composition [13]–[15], or demineralized bone matrices (DBMs) matching the organic composition [16]–[18]. Despite the fact that these materials have demonstrated satisfying cartilage and bone forming capacities, their mechanical properties may not always be optimal for implanting in load bearing sites. In particular, for the bony site under high loads, ceramics are often too brittle and can be subject to fracture, whereas DBMs are very flexible and may require the patient to be temporarily immobilized. A possible solution to overcome the mechanical drawbacks displayed by ceramics and DBMs is to combine and integrate them with a polymeric matrix. Studies to date have focused on the incorporation of ceramics and polymers by interspersing the polymeric phase into the ceramic phase–and vice versa–to finally obtain a homogeneous composite scaffold [19]–[22]. However, this often results in scaffolds with poor polymeric-ceramic bonding, limited control over composite processability and mechanical properties, and loss of interface properties. Furthermore, during the interspersion of the two phases some of the pores can be blocked, typically due to the use of solvents, resulting in a reduction of interconnected pores and lack of cell migration [4], [19], [23]. In this respect, rapid prototyping techniques can offer an optimal solution in terms of mechanical properties modulation as they demonstrated to effectively control the structural parameters of 3D scaffolds [23]–[25]. These technologies can process different biomaterials and require minimal or no use of solvents. In addition, they allow the creation of custom-made scaffolds based on patients' image datasets of the damaged area. These characteristics make rapid prototyping a unique tool to develop readily available products in the clinics.

Here, we propose a novel concept to design hybrid scaffolds where the biomaterials are not chemically or physically bonded, but assembled in a single three-dimensional (3D) construct with preserved pore network interconnectivity and interfacial properties. By integrating different rapid prototyping technologies, it was possible to create scaffolds for osteochondral tissue engineering where the chondral scaffold was directly connected to the bone compartment by 3D fiber-deposition (3DF). The bone construct was formed by ceramic particles of designed shapes captured into a flexible polymeric 3DF matrix (3DFM). A DBM gel was infused and the scaffolds freeze-dried resulting in a foamy interspersed phase that acted as a mechanical cushion while providing further bone regeneration properties. The bone scaffold was finally interlocked to an optimized 3DF scaffold for cartilage regeneration [26], [27] through intertwined concentric polymeric fibers mimicking the tidemark area–region delimiting hyaline cartilage from subchondral bone–of the osteochondral natural architecture. The resulting hybrid constructs combined the flexibility of polymers and DBMs with the mechanical strength of ceramics while maintaining the individual osteochondral formation capacities of the single biomaterials assembled. These polymer-ceramic scaffolds were evaluated for their bone and cartilage regeneration potential and were shown to support *in vivo* osteochondral formation. Poly(ethylene oxide−terephthalate)/poly(butylene terephtalate) - (PEOT/PBT) copolymers - were used to fabricate the polymeric matrices. These polyether-esters are biodegradable thermoplastic elastomers, which have favorable physical properties [28]–[30], and a suitable biocompatibility both *in vitro* and *in vivo* [31]–[35]. Biphasic calcium phosphate (BCP) was chosen as a ceramic due to its osteoconductive and osteoinductive properties depending on its physicochemical and microstructural properties [36]–[39].

## Results

**Figure 1:**
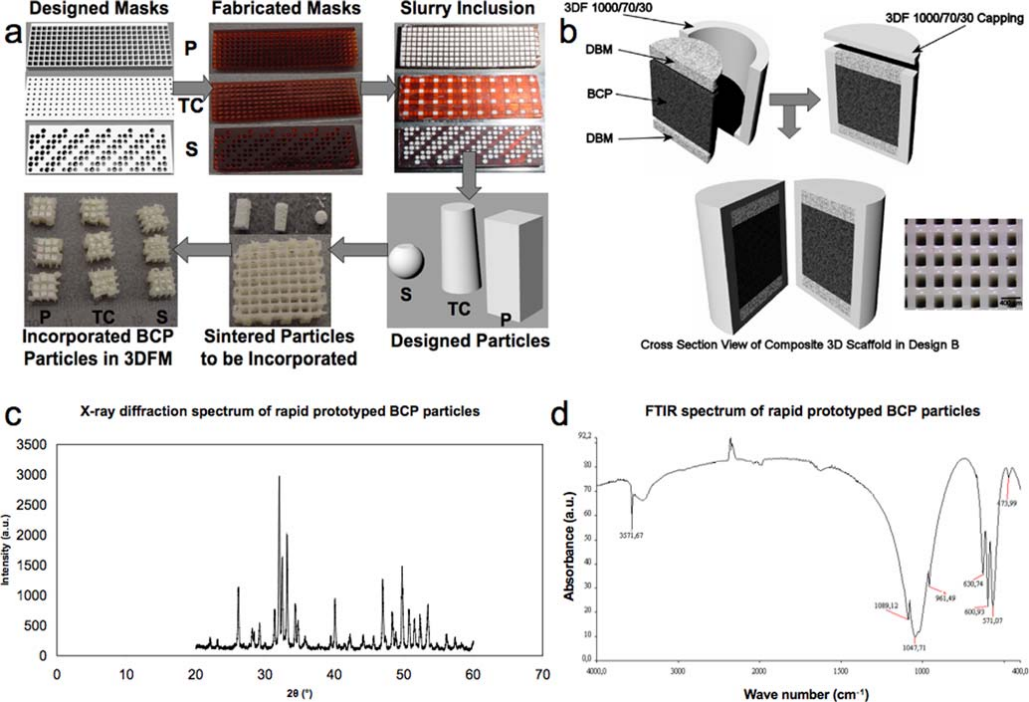
Schematic draw of the scaffold process fabrication for (a) design A and (b) design B. (a) P = Pillar; TC = Truncated Cone; S = Spherical. (b) an optical micrograph of the porous structure of the 3DF hollow cylinder is shown; scale bar: 400 µm. (c) X-ray diffraction pattern and (d) FTIR spectrum of rapid prototyped BCP particles where characteristic peaks are highlighted. Shrinkage following sintering of BCP particles varied from 7±0.7% to 18±1.9%.

The composition of BCP particles was analyzed by X-ray diffraction (XRD) (figure 1c) and was comprised of 24.6% tricalcium phosphate (TCP) and 75.4% hydroxyapatite (HA). Fourier transform infrared spectroscopy (FTIR) confirmed BCP composition, as described elsewhere [38]. DBMs were obtained by acid extraction of the mineralized component of bone, while maintaining the collagen and non-collagenous proteins. Among these proteins, the presence of morphogenic factors is known to confer DBMs osteoinductivity, which contributes to direct the differentiation of local mesenchymal stem cells into the osteogenic lineage [40]–[42].

**Figure 2:**
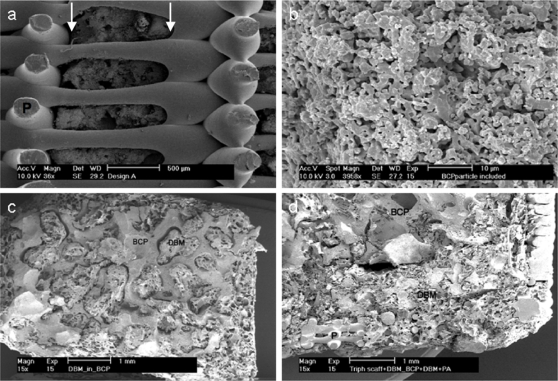
SEM micrographs of designs A (a, b) and B (c, d) integrated 3D hybrid scaffolds. (a) BCP particles inserted in the pores of a 3DF 1000PEOT70PBT30 matrix. (b) microstructure of ceramic particles sintered at T = 1150°C. (c) BCP cylinder infused with DBM after freeze drying. (d) Particular of the whole construct after insertion of the two DBM foamy discs, the infused BCP cylinder in the hollow 3DFM scaffold, and final lyophilization shows the integration of all the components. Scale bar: (a) 500 µm; (b) 10 µm; (c, d) 1 mm. Pillar particles (arrows) are shown here as an exemplification. P = polymer.

Two different design strategies (designs A and B) were considered for the bone compartment of osteochondral scaffolds (figure 1). In the bone compartment of design A, designed particles were captured in the pores of a 3DF polymer matrix (PEOT/PBT 1000/70/30) while in design B a BCP core was surrounded by such a matrix. In both designs the cartilage compartment consisted of the same polymer matrix, but of different composition (300/55/45). The assembled scaffolds were analyzed by scanning electron microscopy (SEM) and evaluated in terms of ceramic-polymer fitting, DBM infusion, and overall construct integrity (figure 2). The weight and volume percentage of BCP included in the 3D PEOT/PBT matrix depended on the particles geometry (supporting information, table S1). Pillar particles were found to better fit in the pores of 3DFM (figure 2a), resulting in the highest BCP weight percentage of 61±3.15% (n = 3). This corresponded to a volume fraction of 27.98±0.81%. SEM revealed a complete infusion of the BCP macro porous structure with DBM (figure 2c). For both the designs, a final infusion in a DBM gel was performed to further integrate the different components assembled in the scaffolds (figure 2d). The final constructs had a pore distribution from 1.43±1.25 µm in the ceramic phase to 1351±2.4 µm in the polymeric matrix and a total porosity of 79.35±1.9% in design A, while a pore distribution from respectively 1.43±1.25 µm to 531±237 µm and a total porosity of 88.95±1.26% in design B (supporting information, table S2).

**Figure 3:**
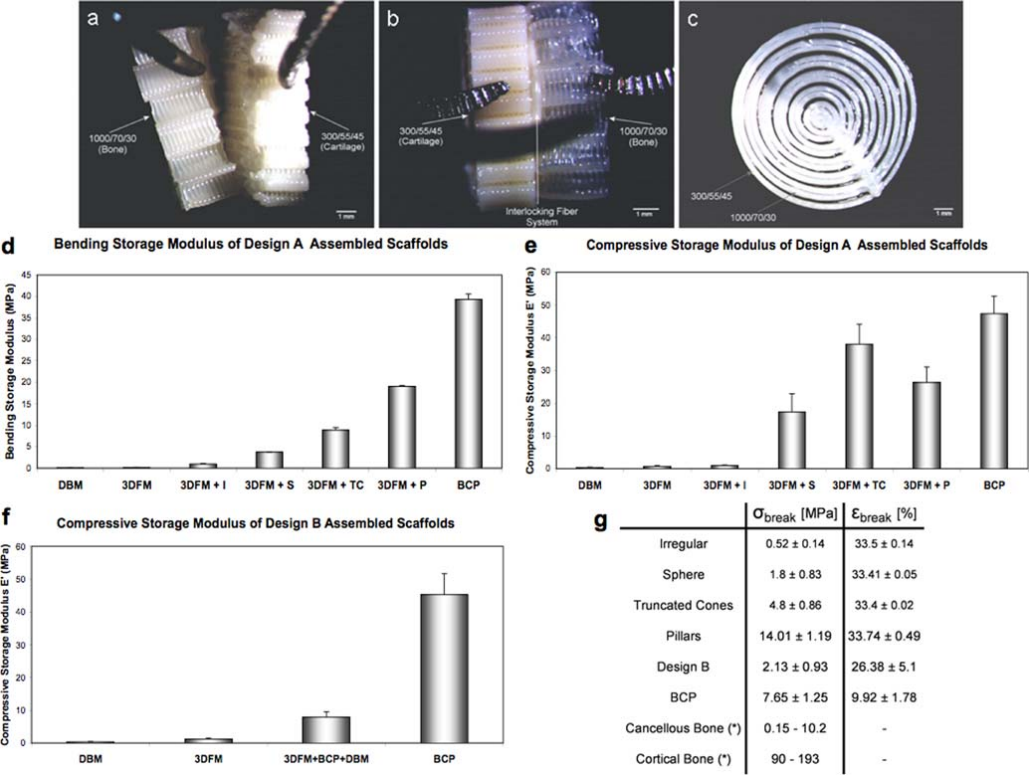
Optical microscopy images of the osteochondral 3D scaffolds. Without the interlocking concentric fiber system (a) both compartments could be separated easily. With the intertwined fibers (c) the osteochondral construct maintained its integrity (b) under mechanical stress. Scale bar: 1 mm. Influence of the scaffold design on the bending (d) and compressive (e, f) storage modulus in designs A (d, e) and B (f). The dynamic stiffness of the single components comprising the hybrid scaffold was also measured for comparison. (g) Stress and strain at break for design A (influence of particle design), design B, and BCP. (*) Strength values for bone are taken from Athanasiou *et al.* [3]. All groups were significantly different from each other (p<0.05). Particle shape legend: I = Irregular; S = Spherical; P = Pillar; TC = Truncated Cone.

Osteochondral constructs with or without an interlocking system were then fabricated by directly depositing the chondral compartment onto the bone scaffold with 3DF (figure 3). Due to the different swelling properties of the two PEOT/PBT compositions (bone = 1000/70/T30–cartilage = 300/55/45), instability at the interface of the two compartments might occur (figure 3a). Therefore, an interlocking system made of intertwined concentric 1000/70/30 and 300/55/45 fibers was deposited at the interface between the bone and the chondral parts of the construct (figure 3b and 3c). SEM analysis revealed a fiber diameter of 170±15 µm, a fiber spacing of 605±12 µm, and a layer thickness of 148±10 µm for the cartilage compartment scaffolds. This corresponded to a porosity of 74±2% and, consequently, to a dynamic stiffness of approximately 13 MPa, as calculated from previous studies [26]. The 3D hybrid scaffolds fabricated for the bone compartment of the osteochondral construct were mechanically characterized by measuring the bending and compressive storage modulus (or dynamic stiffness E′) and the breaking stress and strain. In design A (n = 3), the bending dynamic stiffness of the bare 3DFM scaffold was 0.134±0.035 MPa (figure 3d). When the custom-made BCP particles were press-fitted into the pores of the 3DFM scaffolds the bending stiffness significantly increased to a maximum of 18.99±0.14 MPa for pillar particles (p<0.05), depending on the shape of the particles. Similarly in compression (figure 3e), the dynamic stiffness significantly increased from 0.692±0.16 MPa for bare matrices to 0.935±0.165 MPa for irregular particles, to 37.96±6.14 MPa for truncated conical particles. In design B (figure 3f), the compressive stiffness of the hollow 3DFM cylinder was 1.1±0.34 MPa, while the stiffness of the whole construct was 7.8±1.68 MPa (n = 3, p<0.05). The stress at break was also dependent on the hybrid scaffold design and varied from 0.52±0.14 MPa for irregular particles to 14.01±1.19 MPa for pillar particles. The strain at breaking varied from 9.92±1.78% to 33.74±0.49%, but did not appear to depend on the particle design (figure 3g). DBM and 3DFM alone did not break under compression.

**Figure 4:**
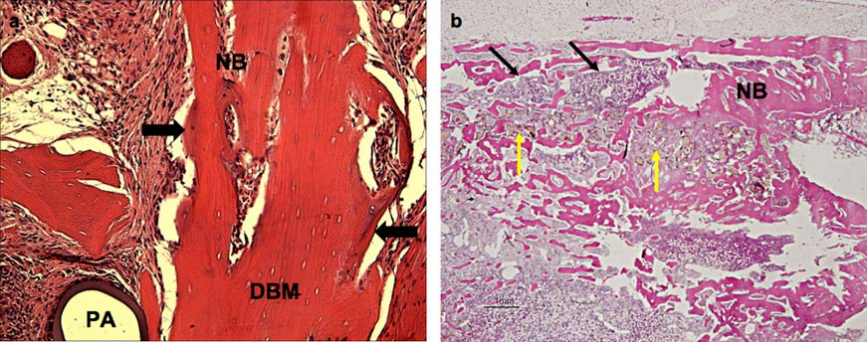
New bone formation in (a) rats and (b) rabbits. (a) Polymeric-DBM 3D scaffolds were implanted for 4 weeks intramuscularly in rats and new bone (arrows) was formed in direct apposition of DBM. (b) When implatend in ulna defects, these scaffolds repaired the defect in 6 weeks with re-establishment of bone marrow (thin black arrows). Polymer degradation was also visible at this time (yellow arrows). NB = new bone; PA = polymeric 3DF scaffold; DBM = demineralized bone matrix.

**Figure 5:**
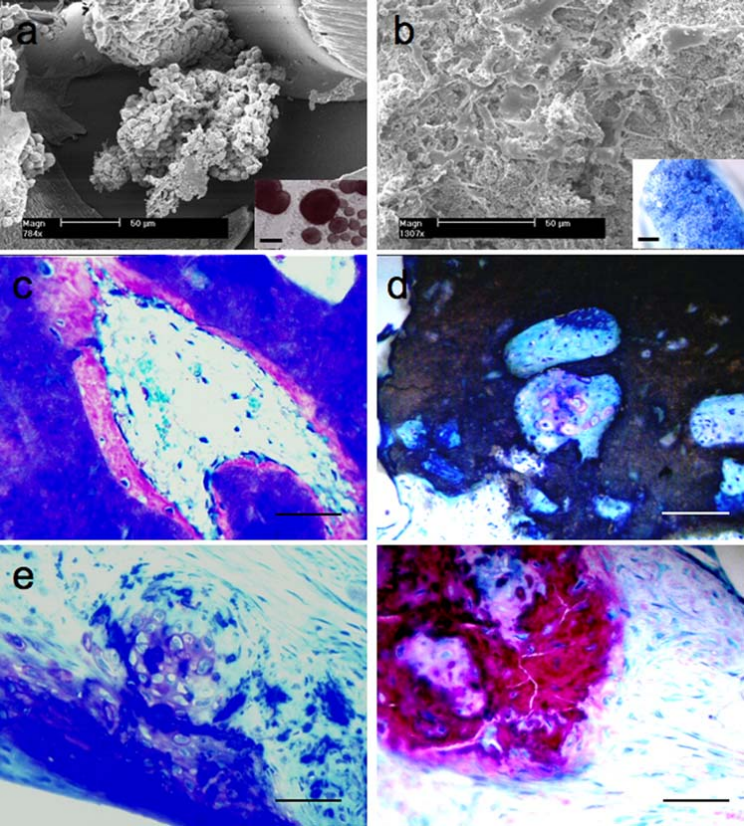
Osteochondral Scaffolds seeded with MSCs before (a, b) and after (c–f) subcutaneous implantation in nude mice. (a) Cell aggregates in the chondral compartment maintained a rounded morphology typical of chondrocytes; insert shows stable aggregate formation after 48–72 hours in chondrogenic media. (b) Cell attached and spread on the BCP particles in the bone part; insert shows methylene blue staining of attached cells on porous pillars. (c) Bone part of the osteochondral construct.: pores were filled with de novo bone (fuchsin red staining). Note the embedded osteocytes and the osteoblasts laying at the outer edge of the mineralized matrix. (d) Occasionally, hypertrophic cells with positive stained matrix could be seen in the pores (thionine). (e) Cartilage part of the osteochondral construct: Cartilage tissue could be observed in the chondral part. Cells exhibit a round, chondrocyte-like morphology, locate din lacunae and surrounded by positive extracellular matrix (thionine staining). (f) Hypertrophic cells in the center of mineralized matrix (fuchsin red staining) and embedded osteocytes could be also occasionally found. Scale bar: (a, b) 50 µm; (c–f) 200 µm; Insert in (a): 250 µm; (b) 600 µm.

As a preliminary study, 3DF polymeric matrices were infused with DBM and assessed *in vivo* for their osteoinductive and osteoconductive properties (figure 4). In an intramuscular rat model (n = 3), new bone formation was observed after 4 weeks in apposition with DBM (figure 4a). In contrast, no bone apposition was detected in polymeric matrices alone, thus highlighting the osteoinductive properties of DBM. To evaluate the full potential of polymeric-DBM biomaterial assembly, scaffolds were implanted in an ulna defect in rabbits (n = 4) for 6 weeks. The bone defect was repaired within this time frame and consistently filled with new bone and marrow (figure 4b). In further efforts to regenerate both cartilage and bone, osteochondral scaffolds as in design A with no DBM infusion were seeded with mesenchymal stem cells (MSC) and evaluated for their cartilage and bone forming capacities subcutaneously in (n = 5) nude mice (figure 5). MSCs were aggregated in chondrogenic medium 2–3 days before seeding and resuspended in the cartilage compartment with Matrigel®, while maintaining a rounded morphology. In the osseous part cells were seeded undifferentiated. Here, they were homogeneously distributed and attached throughout the scaffolds displaying a flat and spread morphology (figures 5a and b). After 25 days of subcutaneous implantation, the two components maintained their structural integrity. Histological analysis revealed de novo bone formation in the bone part (figure 5c).

Tissue generation took place in direct apposition to the ceramic surface. Osseous tissue was composed of a mineralized matrix. Osteocytes could be detected embedded in the matrix and layers of osteoblasts were seen lining the outer edges of the newly formed bone. Bone marrow like tissue characterized by haematopoietic cells, blood vessels and fat could also be observed in most of the implants. In few cases, hyaline cartilage-like islands appeared within the pores of the BCP (figure 5d). In the chondral part histological staining revealed the presence of cartilage like tissue. Cells exhibited a round morphology and were located in lacunae (figure 5e). Mineralized matrix within the chondral part was also noticed. Clearly, hypertrophic cells were still distinguishable in the center of the mineralized nodules suggesting endochondral ossification (figure 5f). Control grafts implanted without cells did not show any evidence of osseous or chondral structures.

## Discussion

In this study, we have shown a novel concept based on biomaterial assembly to design and fabricate 3D osteochondral scaffolds that posses the mechanical flexibility of polymers and DBMs, and the strength of ceramics. The rapid prototyping approach used in the design allowed the creation of scaffolds with a completely interconnected and accessible porous network. Whereas ceramics and DBMs have different mechanical drawbacks, the hybrid composite of these two bone graft substitutes with polymeric matrix has variable stiffness depending on the ceramic particles and on the overall construct design. Strain didn't depend on scaffold design and showed approximately a 3-fold increase with respect to BCP scaffolds, while stress varied from a 14-fold decrease for irregular particles to a 2-fold increase for pillar particles as compared to BCP (figure 3g). The variable increase in stiffness and breaking stress might be linked to the different packing degree of the ceramic in the polymeric matrix, resulting in a progressively higher coupling of the two materials. The increasing fit in the polymer, thus, resulted in a more efficient strengthening of the construct increasing the overall stiffness and the stress at break. At the same time, the presence of DBM and of the polymer introduced a higher flexibility in the constructs due to their intrinsic mechanical properties, causing an increase in the deformation at break. With increasing BCP volume percentage in the hybrid 3D scaffolds, the bending stiffness increased accordingly (figure 4d). This was expected, since BCP is the stiffest component in the assembly. A similar trend was seen for the compressive stiffness, the highest value measured when truncated conical ceramic particles were incorporated in the polymeric matrix (figure 3e). The experimental measured values of the bending stiffness were also in the same range of the calculated stiffness from the Reuss-Voigt model for dispersed particle composites. This might suggest that the assembly of the different biomaterials proposed here can be considered close to a composite when subjected to a flexion load, although it cannot be strictly defined as such. It is clear that the hybrid scaffolds do not mechanically respond as real composites when their mechanical behavior in compression is considered. In this case the theoretical and experimental values do not follow the same power law (supporting information, Figure S1 and Table S3).

As reviewed by Athanasiou *et al.* [3], cancellous bone has a bending modulus varying between 49 MPa and 336 MPa, and a compressive modulus varying between 12 MPa and 900 MPa. Cortical bone has a bending modulus ranging from 5.44 GPa to 15.8 GPa, and a compressive modulus ranging from 4.9 GPa to 27.6 GPa. The variations are related to different bone sources, locations within those sources, and mechanical testing conditions. Cancellous bone has a strength varying between 0.15 MPa and 10.2 MPa, while cortical bone has a strength ranging from 90 MPa to 193 MPa. If the stiffness and stress/deformation at break of the hybrid 3D assembled scaffolds are compared to those of cortical and cancellous bone, constructs with pillar or conical-cylindrical ceramic particles better approach the mechanical behavior of cancellous bone. Yet, the modulation in mechanical properties associated to different ceramic particle geometry in the hybrid 3D scaffolds gives a further degree of freedom to these constructs while maintaining the biological properties of the assembled biomaterials. For example, the use of specific ceramic particles can be envisioned to fit the cancellous bone mechanical properties of each patient in customized applications. Cortical bone still has a much greater stiffness and strength as compared to the scaffolds here presented.

For these reasons, osteochondral scaffolds with pillar BCP particles were selected for in vivo studies and seeded with MSCs, known to be able to differentiate into musculoskeletal tissues under the appropriate stimuli. The use of MSCs is also advantageous regarding clinical applications, since they can be easily isolated by bone marrow aspiration from the iliac crest under partial anesthesia. In this study design, osteogenic differentiation was successfully induced. Noteworthy, cells were not pre-differentiated, but formed bone *in vivo* due to the osteoinductive properties of the BCP particles. Cartilage like tissue within the pores of BCP was also observed. We hypothesize that the enclosure of MSCs supported the regeneration of cartilage-like tissue. Factors such as high cell density, condensation and low oxygen concentration that favor the chondrogenic differentiation of mesenchymal stem cells might have occurred [43]–[47]. It has been shown that PEOT/PBT polymers favor chondrocyte redifferentiation *in vitro* [48], [49]. However, no chondroinductive properties could be detected so far. Therefore, unlike for bone, MSCs intended for cartilage regeneration were pre-cultured in chondrogenic medium for 48–72 hours. Not only cartilaginous tissue was observed, but also mineralized matrix with embedded osteocytes was found in the chondral part. Whereas the presence of cartilage in the bone compartment may be explained through endochondral ossification, it is not yet clear why mineralized matrix was formed in the chondral part of the scaffold. We hypothesize that the presence of blood vessel subcutaneously in mice and their possible ingrowth in the tissue-engineered constructs creates a microenvironment favoring osteogenesis. In an orthotopic location, where an osteochondral scaffold would ultimately be used, the lack of vascular network in the articular cartilage plateau and the proper mechanical loading environment may favor mature cartilage formation with no mineralized matrix in the chondral compartment.

The proposed biomaterial assembly approach to fabricate osteochondral scaffolds through the integration of rapid prototyping technologies brings its novelty as it combines cartilage and bone compartments mechanically matching the natural tissues to be restored with biomaterials that singularly showed to support cartilage and bone tissue formation. The mechanical properties of these constructs could be modulated depending on the assembly design and matched both cartilage and cancellous bone stiffness and strength, while maintaining interface properties. Bone and cartilage were successfully regenerated in the two respective compartments with a single stem cell source. Although further light needs to be shed on the mechanism that brings mineralized matrix formation in chondral scaffolds, these hybrid constructs hold promises as candidates for osteochondral regeneration in one-step surgery procedures.

## Materials and Methods

### Materials Characterization

Poly(ethylene oxide−terephthalate)/poly(butylene terephtalate) (PEOT/PBT) copolymers, solubilized demineralized bone matrix (DBM), and biphasic calcium phosphate (BCP) powder were obtained from IsoTis Orthopaedics S.A. (Bilthoven, The Netherlands). The copolymer composition used in this study were 1000PEOT70PBT30 for the 3DF matrix (3DFM) of the bone compartment and 300PEOT55PBT45 for the cartilage compartment of the osteochondral scaffold where, following an aPEOTbPBTc nomenclature, *a* is the molecular weight in g/mol of the starting PEG blocks used in the copolymerization, while *b* and *c* are the weight ratios of the PEOT and PBT blocks, respectively.

### Design of the Bone Compartment Scaffolds

Tri-phasic scaffolds were fabricated by assembling BCP, 1000PEOT70PBT30, and DBM into monolithic constructs for the bone compartment of the osteochondral scaffold. Two different design schemes (A and B) were chosen for the scaffolds. Design A consisted of fabricating a PEOT/PBT 3DFM that was used as a carrier for BCP rapid prototyped particles, which were press fitted into the scaffold pores. BCP particles were of pillar (side: 1.6 mm; height: 4.3 mm), truncated cone (large base diameter: 2 mm; small base diameter: 1.6 mm; height: 4 mm), spherical (diameter: 1.8 mm) and irregular (between 1.4 mm and 2 mm in their maximum dimension) shapes (figure 1a). More spherical and irregular particles were press fitted in the pores of the 3DFM scaffolds until covering the total thickness of the polymeric matrix. Different ceramic particle shapes were considered to assess the optimal amount of included BCP and the influence of the particle geometry on mechanical properties, while maintaining the flexibility of the construct. The 3DFM scaffold had a block shape, with a square base of 10 mm, and a height of 3.15 mm. Since 1000PEOT70PBT30 is known to swell in an aqueous environment [28]–[30], the scaffolds were under dimensioned to match exactly the BCP designed particles in a wet milieu.

In design B, a BCP cylinder (5 mm in diameter by 3 mm in height) was pre-soaked in a DBM gel (2.6% w/v in a 4.75% v/v methanol in demineralized water solution) and subsequently lyophilized. The cylinder was “sandwiched” by two discs of DBM foam (5 mm in diameter by 1 mm in height) and the construct was inserted in a hollow cylindric container of PEOT/PBT made by three-dimensional fiber deposition (figure 1b). The polymeric hollow cylinder had an outer diameter of 6 mm, a wall thickness of 1 mm and a height of 6 mm. In both design A and B, the whole constructs were immersed again in the DBM gel and freeze-dried. Figure 1 illustrates the principle of the assembling procedure.

### Fabrication of the Bone Compartment Scaffolds

#### Polymeric 3DFM Matrix

1000PEOT70PBT30 3DFM scaffolds were manufactured with a Bioplotter device (Envisiontec GmbH, Germany), essentially an XYZ plotter device as previously described [30]. The device was modified to extrude highly viscous polymeric fibers. The polymer was put in a stainless steel syringe and heated at a temperature T = 190°C through a thermoset cartridge unit, fixed on the “X”-mobile arm of the apparatus. When the molten phase was achieved, a nitrogen pressure of 5 bars was applied to the syringe through a pressurized cap. The scaffold models were loaded on the Bioplotter computer aided manufacturing (CAM, PrimCAM, Switzerland) software and deposited layer by layer, through the extrusion of the polymer on a stage as a fiber. The deposition speed was set to 300 mm/min. Scaffolds were then characterized by the fiber diameter (through the nozzle diameter), the spacing between fibers in the same layer, the layer thickness and the configuration of the deposited fibers within the whole architecture. In design A, the nozzle used was a stainless steel Luer Lock needle with internal diameter (ID) of 400 µm, shortened to a length of 16.2 mm. The fiber spacing was set to 1650 µm, while the layer thickness to 225 µm. A 0-90 scaffold architecture was chosen, where fibers were deposited with 90° orientation steps between successive layers. In design B, the hollow cylindrical scaffold was fabricated with a similar nozzle with respect than design A, but with a smaller ID of 250 µm. The fiber spacing was decreased to 600 µm, and the layer thickness to 150 µm, while the fiber deposition architecture was maintained as a 0-90 architecture.

#### Ceramic Particles

Porous BCP designed particles were fabricated by an indirect rapid prototyping technique. First negative masks were designed with computer aided design (CAD) software (Rhinoceros®) and fabricated with an acrylic photopolymerizable resin by photolithography (PreFAB, Envisiontec, Germany). The masks were then filled with a BCP slurry made by adding 32.8 grams of calcinated BCP powder (20 hours in an oven at 1000°C), 14.2 grams of not calcinated BCP powder, 20 grams of demineralized water, 1.2 grams of methylcellulose solution (2% w/v methylcellulose in demineralized water), 2.2 grams of ammonia, and 14.1 grams of 300–500 µm sieved naphthalene particles, resulting in a 30% macro porosity of the BCP particles. The components were blended and vigorously stirred with a mixer for approximately 30 minutes, until a homogeneous slurry was obtained. The BCP particles were then obtained by debonding and sintering in a furnace (Nabertherm, Germany) at T = 1150°C. Irregular BCP particles with an average size between 1.4 mm and 2 mm were also used. These latter particles were fabricated by hydrogen peroxide foaming, as described elsewhere [14]. BCP particles were press fitted in the pores of the 3DF matrix by exploiting the swelling behavior of 1000PEOT70PBT30. The polymeric matrix was left in demineralized water for 24 hours to allow swelling prior to insertion of the BCP particles.

#### 3D Hybrid Scaffold Assembly

DBM gel was obtained by mixing 2 grams of DBM powder into 80 grams of methanol solution (4.75% volume/volume in demineralized water). DBM foams were fabricated by placing the gel in square molds and freeze-drying (Virtis 25 SRC, The Netherlands). The scaffolds were finally immersed in the DBM gel and placed for 1 hour under vacuum (0.01 mbar) to let the gel impregnate the porous polymeric matrix and the BCP particles. After infiltration the final construct was freeze-dried to obtain a porous DBM matrix infiltrating and surrounding the scaffolds. This resulted in a final hybrid scaffold thickness of approximately 6 mm for each design.

#### 3D Osteochondral Scaffolds Fabrication

The 300PEOT55PBT45 cartilage compartment of the osteochondral scaffold was deposited directly on top of the bone assembled 3D hybrid scaffold by 3DF. In a similar process to what previously explained, the polymer was placed in the extrusion syringe and heated through the thermoset cartridge to reach its molten state at T = 210°C. A nitrogen pressure of 5 bars was applied to extrude the polymeric fibers from a nozzle with an ID of 250 µm and a length of 16.2 mm. The fiber spacing was set to 600 µm, and the layer thickness to 150 µm, while the fiber deposition architecture was maintained as a 0-90 architecture. The fibers were deposited at a speed of 230 mm/min. Having the two PEOT/PBT compositions (bone = 1000PEOT70PBT30–cartilage = 300PEOT55PBT45) different swelling properties, osteochondral scaffolds with and without an interlocking fibrous system were fabricated to assess the interface resistance of the construct. The interlocking system was made of intertwined concentric 1000PEOT70PBT30 and 300PEOT55PBT45 fibers at the interface between the bone and the chondral parts of the construct. The concentric fibers were extruded from needles with ID and length as above described. The fiber spacing was set to 800 µm, and the layer thickness to 135 µm. The fiber spacing was chosen to obtain a precise interlock of the fibers after swelling. The resulting osteochondral scaffolds had a diameter of 6 mm and a height of approximately 8 mm.

#### Scaffolds Characterization

Cylindrical plugs of 6 mm in diameter by 6 mm in height were taken as samples for characterization (n = 3). The constructs were analyzed with an optical microscope (OM) to assess their integrity over time and by scanning electron microscopy (SEM) analysis with a Philips XL 30 ESEM-FEG. Samples were gold sputter coated (Carringdon) before SEM analysis. The porosity of the 3DF cartilage scaffold and of the bone scaffold in its separated components and as a final whole construct was experimentally measured by analyzing the mass and the volume of each structure, as:
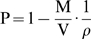
(1)where M and V are the measured mass and volume of the scaffolds components, while ρ is the specific density of the materials (1.25 g/cm^3^ for 1000PEOT70PBT30, 3.15 g/cm^3^ for BCP, and assumed to be 1 g/cm^3^ for DBM, since the mineral component was extracted). The composition of BCP particles was analyzed by x-ray diffraction (XRD) (Rigaku Miniflex, China) and Fourier Transform Infrared analysis (FTIR) (Spectrum 1000, Perkin Elmer, USA).

For the bone hybrid construct, the weight and volume percentage of BCP included in each scaffold was also measured as the ratio between the BCP particles weight and volume and the weight and volume of the final construct, respectively (Supporting Information, Table S1).

#### Mechanical Characterization of the Bone Hybrid Scaffolds

A DMA instrument (Perkin Elmer 7e) was used to evaluate the bending and compressive dynamic stiffness of the 3D assembled scaffolds of the bone compartment and of the single biomaterials used (n = 3). In the dynamic bending test, three slabs of 15 mm in length by 5 mm in width by 6 mm in height were used as samples. In the case of the hybrid constructs, only scaffolds from design A were tested in the bending configuration as the intrinsic construction of scaffolds in design B did not allow for their bending characterization. A 3-point bending test was chosen for the characterization. Scaffolds were loaded with a dynamic force varying from 350 mN to 450 mN. A ramp of 5 mN/min at a constant frequency of 1Hz was applied. A lower range of forces was applied with respect to the compressive dynamic test to prevent sample deformation that impinged with the test setting.

In the compressive dynamic test, for each hybrid design and for each single biomaterial used three cylindrical samples of 6 mm in diameter by approximately 6 mm in height were tested. Cylindrical fixtures were chosen to test the specimens and evaluate their behavior as a whole structure along their compression axis, in the “z-direction”. Scaffolds were loaded with a dynamic force varying from 3.5N to 4.5N. A ramp of 50 mN/min at a constant frequency of 1Hz was used. In the two test configurations, the dynamic stiffness, or storage modulus E′, was calculated in the elastic region of the composites. The theoretical modulus as proposed by the Reuss-Voigt model for a composite was also calculated [50]. In this case we assumed the ceramic particles as mainly oriented along the longitudinal direction where compression occurs. The modulus can then be calculated as:
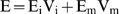
(2)where E is the modulus of the final construct, E_i_ and V_i_ are the modulus and the volume fraction of the inclusions (here considered as the BCP particles), while E_m_ and V_m_ are the modulus and the volume fraction of the polymeric matrix.

The stress and deformation at break were measured with a Zwick Z050 mechanical testing apparatus (Zwick, Germany), in a failure test under compression with a crosshead speed of 1 mm/min.

#### Bone marrow isolation and cell expansion

Goat bone marrow cells (gBMCs) were isolated and culture expanded as described previously [51]. Briefly, bone marrow aspirates from the iliac wing of Dutch milk goats were plated in tissue culture flasks (5×10^5^ nucleated cells/cm^2^) and cultured in expansion medium containing α-Modified eagle medium supplemented with 15% fetal bovine serum (FBS), 1% Penicillin/Streptomycin, 0.1 mM ascobate-2- phosphate acid and 2 mM L-Glutamine until reaching 80% confluence. gBMCs were harvested using 0.25% trypsin-EDTA, counted and replated at 1000 cells/cm^2^. Cells were cultured in monolayer in a humidified atmosphere with 5%CO_2_ at 37°C. Medium was changed every 2–3 days. When reaching 80% confluence again, cells were trypsinated, washed twice in phosphate buffer saline (PBS) solution and counted using a Burker turk counting chamber.

#### Cell seeding

Osteochondral grafts were incubated in expansion medium 48 h prior to the implantation. Four samples without cells served as controls. For the cell-based constructs two different methods were used to seed the two different parts of the osteochondral graft. For the osseous part, gBMCs were seeded at a density of 2.5×10^6^ cells/graft onto the BCP component 4 h prior to the implantation. For the chondral component 48 h prior to the implantation cells were incubated in a 24-well plate at a cell density of 1×10^6^ cells/well in Dulbecco's modified eagle medium supplemented with 1% penicillin/streptomycin, 1% ITS^+^ (insulin, transferring, selenious acid), 100 nM dexamethasone, 50 µg/ml ascorbic acid-2-phosphate, 100 µg/ml sodium pyruvate, 40 µg/ml proline and 10ng/ml transforming growth factor (TGF) β1 (R&D Systems, Abington, UK). Prior to implantation cells of five wells were collected, spun down at 300g for 30 seconds and supernatant was removed. Cells were resuspended in 50 µl Matrigel® (BD Bioscience, Alphen aan den Rijn, The Netherlands) and placed into the pores of the chondral part of the graft (total of 5×10^6^ cells/graft). Samples were incubated for 20 minutes at 37°C in order to let a gel be formed.

#### Implantation

Animals were housed at the Central Laboratory Animal Institute (Utrecht University, The Netherlands). All animal experiments have been approved by the local Animal Care and Use committee (DEC) and performed in adherence to the local and national ethics guidelines. The animals were acclimated for a minimum of 5 days under the same conditions as the actual test. The animals were housed in micro isolation polycarbonate cages with sterile contact bedding, supplied with irradiated, certified commercial feed and autoclaved, potable water.

To assess osteoinductivity, hybrid scaffolds were implanted intramuscularly in (n = 3) 2–3 months old male rats (Wistar, Charles River). Each rat received one intramuscular implants in the leg. The animals were anesthesized and prepared for surgery. With sharp and blunt dissection, a pocket was created in the femur bicep (hamstring) muscle. The scaffold was placed into the pocket and the muscle pocket and skin was suture closed. The animals were recovered from the anesthesia and retuned to their cages. All animals were observed daily for abnormal clinical signs. After 4 weeks the animals were sacrificed and the implants removed.

To assess osteoconductivity, hybrid scaffolds were implanted in an ulna defect in (n = 4) of six-month-old female New Zealand White rabbits. Animals were kept in separate cages, fed a standard diet, and allowed to move freely during the study. At surgery, the right forearms were shaved and draped in a sterile fashion under general anesthesia with intravenous sodium pentobarbital (30 mg/kg of body weight). An antibiotic (netilmicin 4 mg/kg of body weight) was administered perioperatively. A 1.5 cm segmental bone defect was created in the diaphysis of the right ulna using an oscillating saw under irrigation with sterile saline solution. The periosteum attached to the resected bone segment was removed, and the defect site was irrigated with sterile PBS. After press-fit insertion of the implants the fascia, subcutaneous tissues and skin were closed using absorbable sutures and non-absorbable sutures, respectively. Animals were allowed full weight-bearing activity immediately following the surgery. Implants were evaluated after 6 weeks.

Tissue engineered osteochondral grafts and controls were implanted into six weeks old male immunosufficient mice (HdCpb:NMRI-nu, Harlan). Animals (n = 5) were operated under aseptic conditions. After subcutaneous injection of 0,05 mg/kg Temgesic for analgesia the mice were put under general inhalation anesthesia using Isoflurane. Two subcutaneous pockets were created on the dorsum of each mouse by blunt dissection. One osteochondral graft was inserted per pocket. Evaluation was assessed after 25 days.

#### Histological analysis

In rat and rabbit studies, scaffolds were removed from the implantation site, fixed in 10% formalin and dehydrated in a graded ethanol series. Samples were then embedded in polymethylmethacrylate (Sigma, The Netherlands). Histological sections of 7 µm were cut using a sawing microtome (Leica, Germany) and stained with haematoxylin and eosin.

In the mice study, 25 days after implantation mice were euthanized by CO_2_ asphyxiation. The implants were carefully removed and fixed in 1,5% glutaraldehyde in 0,14 M sodium cacodylate buffer for 24 h at 4°C. Following dehydration by graded ethanol series specimens were embedded in polymethylmethacrylate. Histological sections of 10 µm were made using the same microtome and stained with 1% methylene blue and 0,3% basic fuchsin to visualize bone formation or 0,04% thionine to distinguish cartilage like tissue formation.

### Statistical Analysis

Statistical Analysis was performed using a Student's t-test, where the confidence level was set to 0.05 for statistical significance. Values in this study are reported as mean and standard deviation.

## Supporting Information

Table S1 BCP weight percentage included in the assembled scaffolds depending on particle design.
(0.28 MB TIF)

Table S2 Pore size and porosity distribution of the single components and of the final scaffold constructs.
(0.32 MB TIF)

Table S3 Comparison between the experimental and the theoretical (Reuss-Voigt model) values of the storage moduli of 3D scaffolds with custom-designed assembled BCP particles.
(0.33 MB TIF)

Figure S1 Influence of the BCP volume fraction on the bending, compressive, and Reuss-Voigt moduli. Bending modulus: r^2^ = 0.96; Reuss-Voigt modulus: r^2^ = 0.99; compressive modulus: r^2^ = 0.87.
(0.66 MB TIF)
