# Supplementary figures and images for: Regenerating Articular Tissue by Converging Technologies
Source: PLoS One. 2008 Aug 21;3(8):e3032. doi: 10.1371/journal.pone.0003032 (PMC2515637; doi:10.1371/journal.pone.0003032)

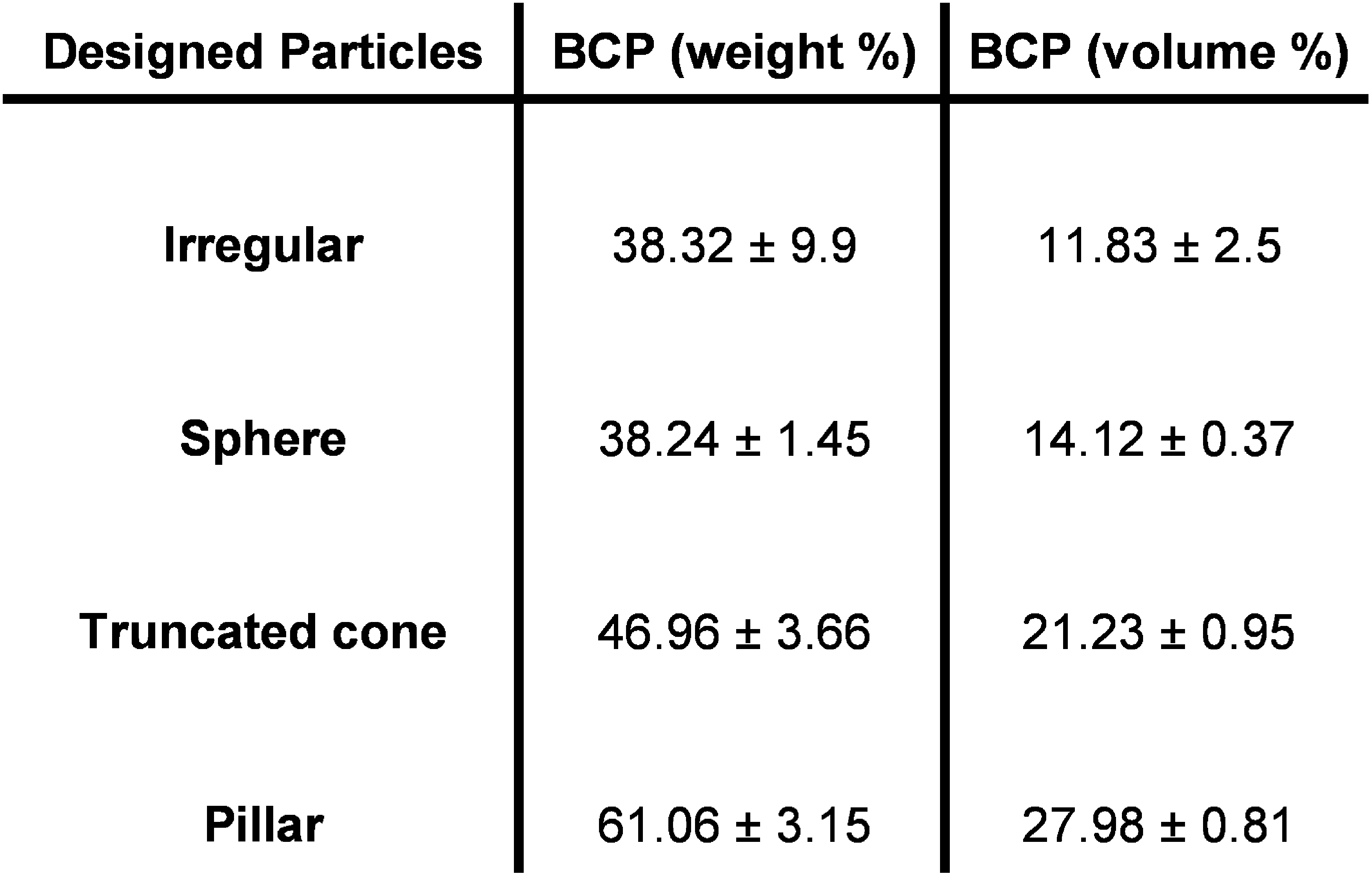

Supplement: Table S1 — BCP weight percentage included in the assembled scaffolds depending on particle design. (0.28 MB TIF) [file pone.0003032.s001.tif]

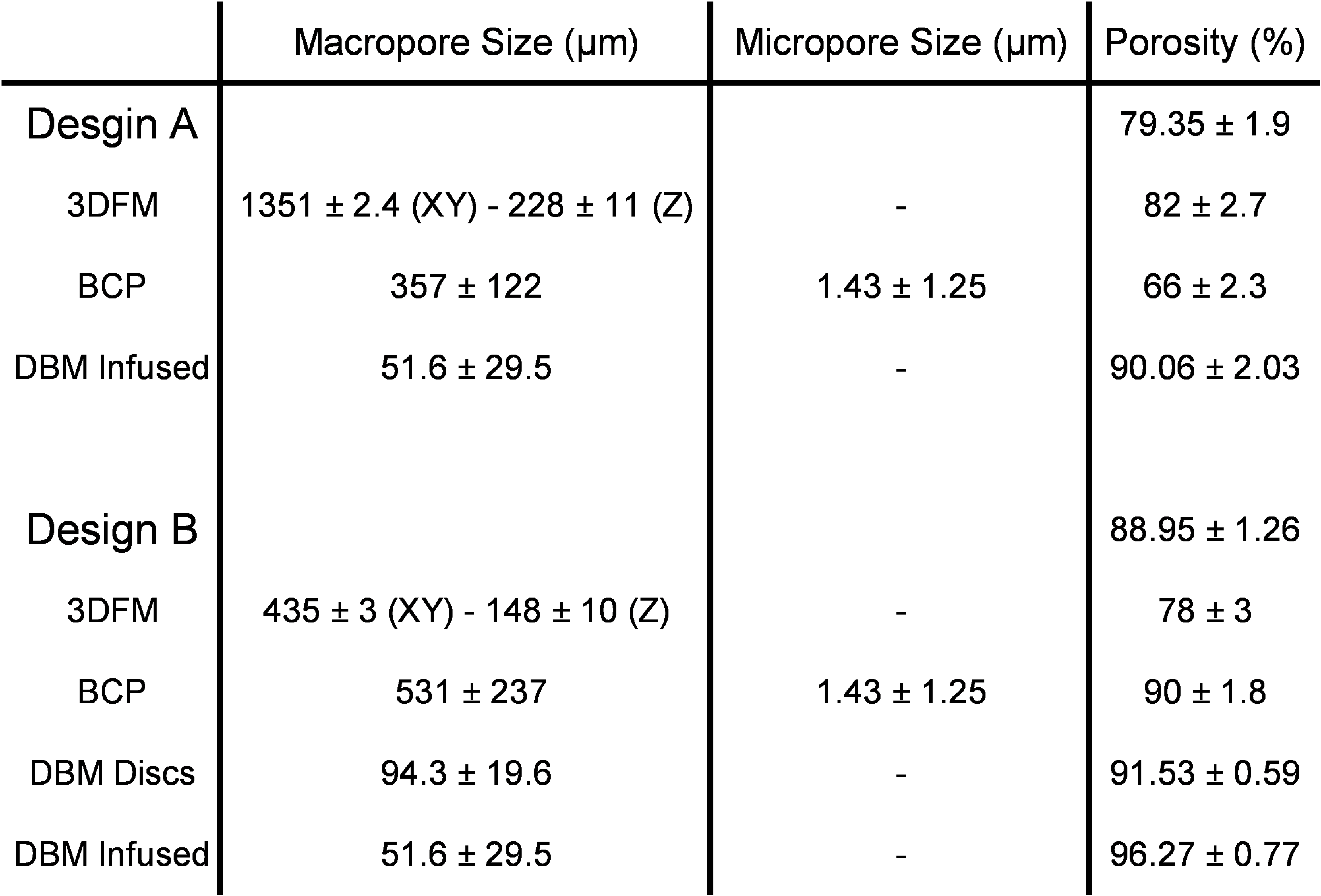

Supplement: Table S2 — Pore size and porosity distribution of the single components and of the final scaffold constructs. (0.32 MB TIF) [file pone.0003032.s002.tif]

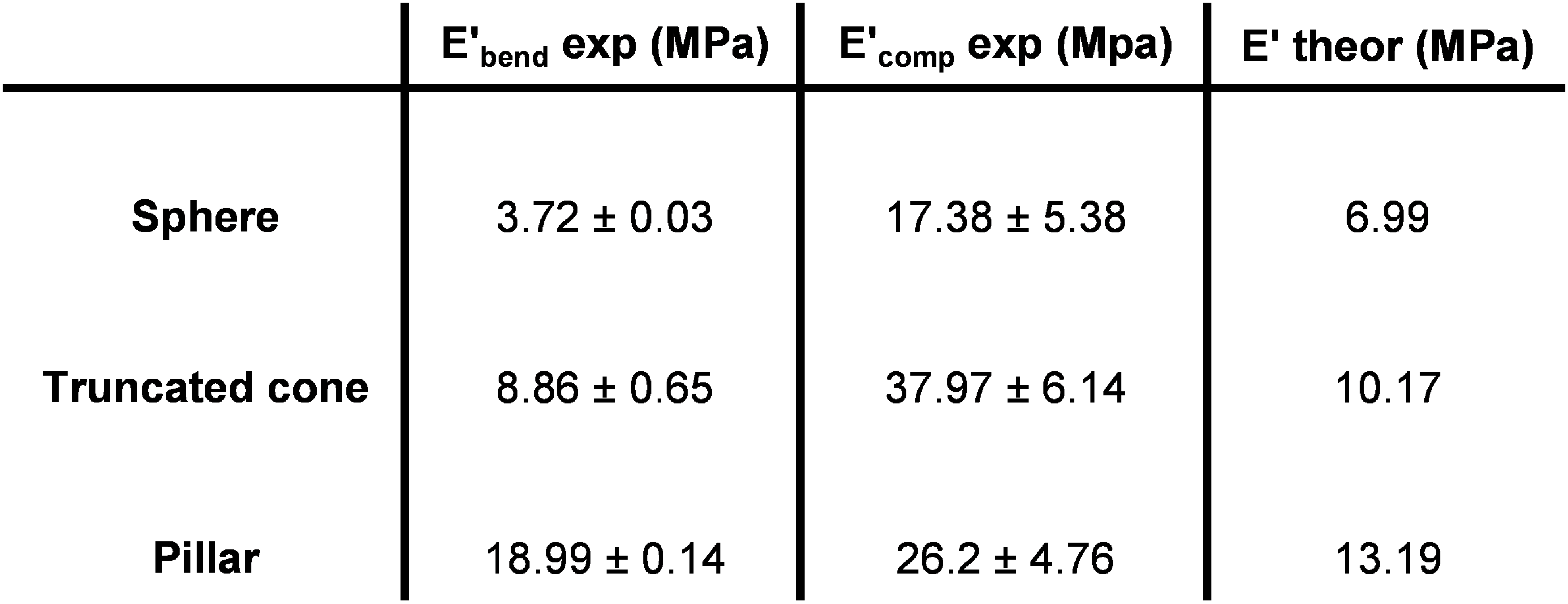

Supplement: Table S3 — Comparison between the experimental and the theoretical (Reuss-Voigt model) values of the storage moduli of 3D scaffolds with custom-designed assembled BCP particles. (0.33 MB TIF) [file pone.0003032.s003.tif]

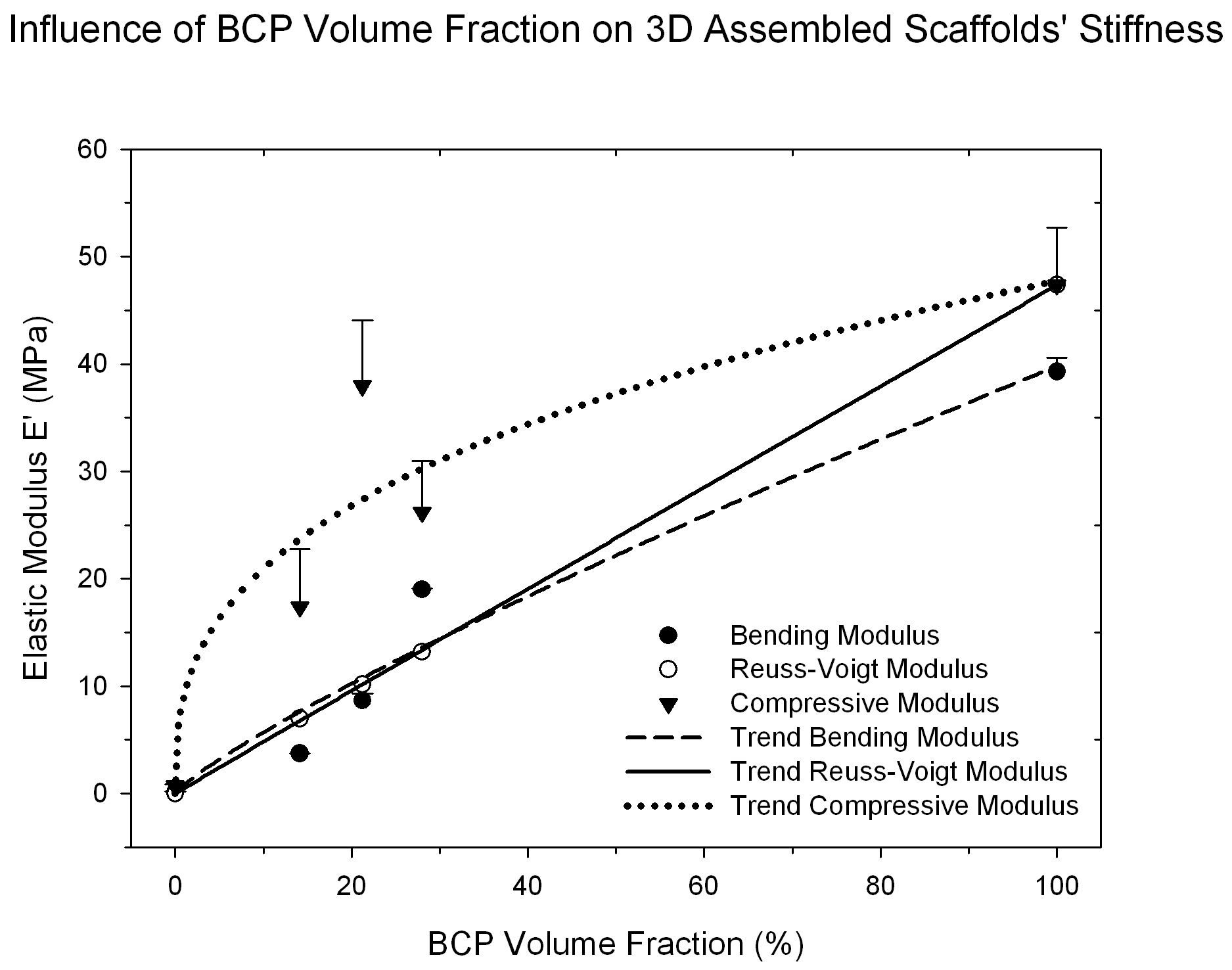

Supplement: Figure S1 — Influence of the BCP volume fraction on the bending, compressive, and Reuss-Voigt moduli. Bending modulus: r2 = 0.96; Reuss-Voigt modulus: r2 = 0.99; compressive modulus: r2 = 0.87. (0.66 MB TIF) [file pone.0003032.s004.tif]
